# The relationship of C-reactive protein/interleukin-6 concentrations between serum and synovial fluid in the diagnosis of periprosthetic joint infection

**DOI:** 10.1186/s13018-021-02880-x

**Published:** 2021-12-20

**Authors:** Bao-Zhan Yu, Rui Li, Xiang Li, Wei Chai, Yong-Gang Zhou, Ji-Ying Chen

**Affiliations:** 1 Department of Orthopaedics, Chinese PLA General Hospital, 28 Fuxing Rd, Beijing, 100853 People’s Republic of China; 2 Department of Orthopaedics, No.2 Hospital of Baoding, Baoding, Hebei People’s Republic of China

**Keywords:** C-reactive protein, Interleukin-6, Periprosthetic joint infection, Serum, Synovial fluid, Diagnosis

## Abstract

**Background:**

The relationship of C-reactive protein (CRP)/interleukin-6 (IL-6) concentrations between serum and synovial fluid and whether synovial CRP/IL-6 testing in addition to serum CRP/IL-6 testing would result in a benefit in the diagnosis of periprosthetic joint infection (PJI) deserves to be investigated.

**Methods:**

From June 2016 to July 2019, 139 patients were included in the study. Synovial CRP and IL-6 were tested by ELISA. The serum CRP and IL-6 were obtained from medical records. The definition of PJI was based on the modified Musculoskeletal Infection Society (MSIS) criteria. The relationship of serum and synovial CRP and IL-6 and the value of each index in the diagnosis of PJI were evaluated.

**Results:**

The receiver operating characteristic (ROC) curves showed that synovial IL-6 had the highest area under the curve (AUC) at 0.935, which was followed by synovial CRP, serum IL-6 and serum CRP 0.861, 0.847 and 0.821, respectively. When combining serum CRP and synovial CRP to diagnose PJI, the AUC was 0.849, which was slightly higher than the result obtained when using serum CRP alone. In contrast, when combining serum IL-6 and synovial IL-6 to diagnose PJI, the AUC increased to 0.940, which was significantly higher than that obtained using serum IL-6 alone.

**Conclusion:**

The synovial IL-6 has the highest diagnostic accuracy for PJI. However, inferring the level of CRP/IL-6 in the synovial fluid from the serum level of CRP/IL-6 was not feasible. Synovial CRP testing did not offer an advantage when combined with an existing serum CRP result to diagnose PJI, while additional synovial IL-6 was worthy of testing even if there was an existing serum IL-6 result.

## Introduction

Periprosthetic joint infection (PJI) is a serious complication after total knee or hip arthroplasties and is associated with a large economic burden on healthcare systems and increased morbidity and mortality [1, 2]. It is important to make an early and accurate diagnosis for the treatment of periprosthetic infection. However, the diagnosis of PJI is a challenging task for orthopedic surgeons [3]. The diagnosis often requires a comprehensive judgment based on a series of tests [4].

Among the various kinds of tests, using serum markers would be simpler and more practical than other methods. Serum markers are often used as a first-line screening method. Numerous serum markers, such as erythrocyte sedimentation rate (ESR), C-reactive protein (CRP), interleukin-6 (IL-6), D-dimer and fibrinogen, are reported to have good diagnostic accuracy in the diagnosis of PJI [4–7]. However, because serum indicators are susceptible to systemic states, they cannot be the only evidence for the diagnosis of PJI [8]. Therefore, synovial fluid tests such as white blood cell [WBC] count and differential, interleukin-6 (IL-6), a-defensin are often needed to further clarify the diagnosis.

Because of the extra cost for each test and the limited volume of synovial fluid aspirated from some patients, choosing the proper synovial marker for further confirmation is crucial. The basic requirement of the synovial test is that the synovial test alone is more accurate than the serum test [9, 10]. Ideally, a combination of serum and synovial tests would yield a higher diagnostic value.

Among various kinds of synovial markers, C-reactive protein (CRP) and interleukin-6 (IL-6) are two synovial markers that are often used as serum markers in the diagnosis of PJI. Catterall et al. [11] postulated that the diffusion of serum CRP into the joint could be responsible for an elevated synovial fluid CRP level when CRP is elevated systemically. Thus, there is a possibility that the levels of the markers in serum could be used to predict the levels of the markers in synovial fluid. It is worth exploring the CRP/IL-6 concentrations between serum and synovial fluid. In other words, whether further synovial CRP/IL-6 testing could result in a PJI diagnostic benefit in addition to an already known serum CRP/IL-6 result deserves to be investigated.

Therefore, the purpose of this study was to explore the relationship between the serum and synovial levels of IL-6 and CRP in the diagnosis of PJI and to investigate whether synovial CRP or IL-6 could have diagnostic value in addition to the serum levels in the diagnosis of PJI.

## Methods

This study was approved by our institution’s review board. Between June 2016 and July 2019, a total of 151 synovial fluid samples were successively collected and stored in a − 80 ℃ freezer after centrifugation. All of these samples were aspirated from patients with any signs or symptoms indicating the possibility of infection after total knee or hip arthroplasties, such as acute onset of pain or persistent pain after surgery, an elevated ESR and/or CRP level, or implant failure within 5 years after primary arthroplasty without any reasonable explanation.

Among the patients who provided the 151 synovial fluid samples, one patient did not have recorded serum levels of CRP, five patients did not have recorded serum levels of CRP and IL-6, and six patients did not have recorded serum levels of IL-6. Ultimately, 139 patients were included in the study. The study group consisted of 46 men and 93 women with a mean age of sixty-five years (range, 34–88 years) at the time of the diagnostic aspiration. Fourteen patients had a diagnosis of systemic inflammatory disease, including rheumatoid arthritis (eleven), ankylosing spondylitis (two), and dermatomyositis (one). Three patients were taking a medication that modulates the immune system.

Serum CRP, IL-6 and other related tests, such as ESR, were part of our routine work-up for suspected PJI cases, and the corresponding blood samples were sent to the medical laboratory center for testing as soon as possible. In addition to the tests mentioned above, the synovial fluid white blood-cell (WBC) count, the percentage of polymorphonuclear cells or neutrophils, the leukocyte esterase test, and culture and histological analyses were assessed to determine PJI. The definition of PJI was based on the modified Musculoskeletal Infection Society (MSIS) criteria [12]. All data mentioned above were obtained from electronic medical records.

The synovial fluid samples were taken from the remainder of routine examination, which they were centrifuged (SCILOGEX D3024, 6600 RPM) for three minutes. The isolated supernatant was aliquoted into sterile tubes and stored at -80 °C. The synovial CRP and IL-6 levels were tested with enzyme-linked immunosorbent assay (ELISA) kits by a third-party laboratory (Beijing Protein Innovation Co., Ltd.). All assays were carried out according to the manufacturer’s instructions (Human CRP Quantikine ELISA Kit; Human IL-6 QuantiGlo ELISA Kit, RD).

### Statistical analysis

Statistical analyses were performed using Empower (R) (X&Y Solutions) and R software (The R Foundation), and scatterplots were drawn with GraphPad Prism (version 7.00 for Windows; GraphPad Software, Inc.). The continuous variables were analyzed with the Mann–Whitney test, and categorical variables were analyzed with chi-square tests. A *P* value of < 0.05 was considered significant. Receiver operating characteristic (ROC) curves were used to evaluate the diagnostic value of each index. The optimal threshold for each marker as a diagnostic tool for PJI was determined using the Youden index. Linear regression was used to evaluate the relationship of serum and synovial indexes. Diagnostic prediction models were used for the comparisons between two diagnostic strategies.

## Results

Among the 139 included patients, 62 patients were diagnosed with PJI, and 77 patients were not diagnosed with PJI based on the modified MSIS criteria. The characteristics of the PJI group and non-PJI group are shown in Table 1.

**Table 1:** Characteristics of the PJI and non-PJI groups based on the modified MSIS criteria

Variables	PJI (*n* = 62)	Non-PJI (*n* = 77)	*P* value
Age† (yr)	65.85 (61–71)	64.60 (59–71)	0.422
Sex*			
Male	28 (45.16%)	18 (23.38%)	0.007
Female	34 (54.84%)	59 (76.62%)	
BMI†‡ (kg/m^2^)	25.30 (23.21–27.36)	25.49 (23.14–28.06)	0.809
Joint*			0.551
Hip	18 (29.03%)	26 (33.77%)	
Knee	44 (70.97%)	51 (66.23%)	

^*^The values are given as the number of cases, with the percentage in parentheses

^†^The values are given as the median, with the interquartile range in parentheses

^‡^*BMI* body mass index

**Fig. 1:**
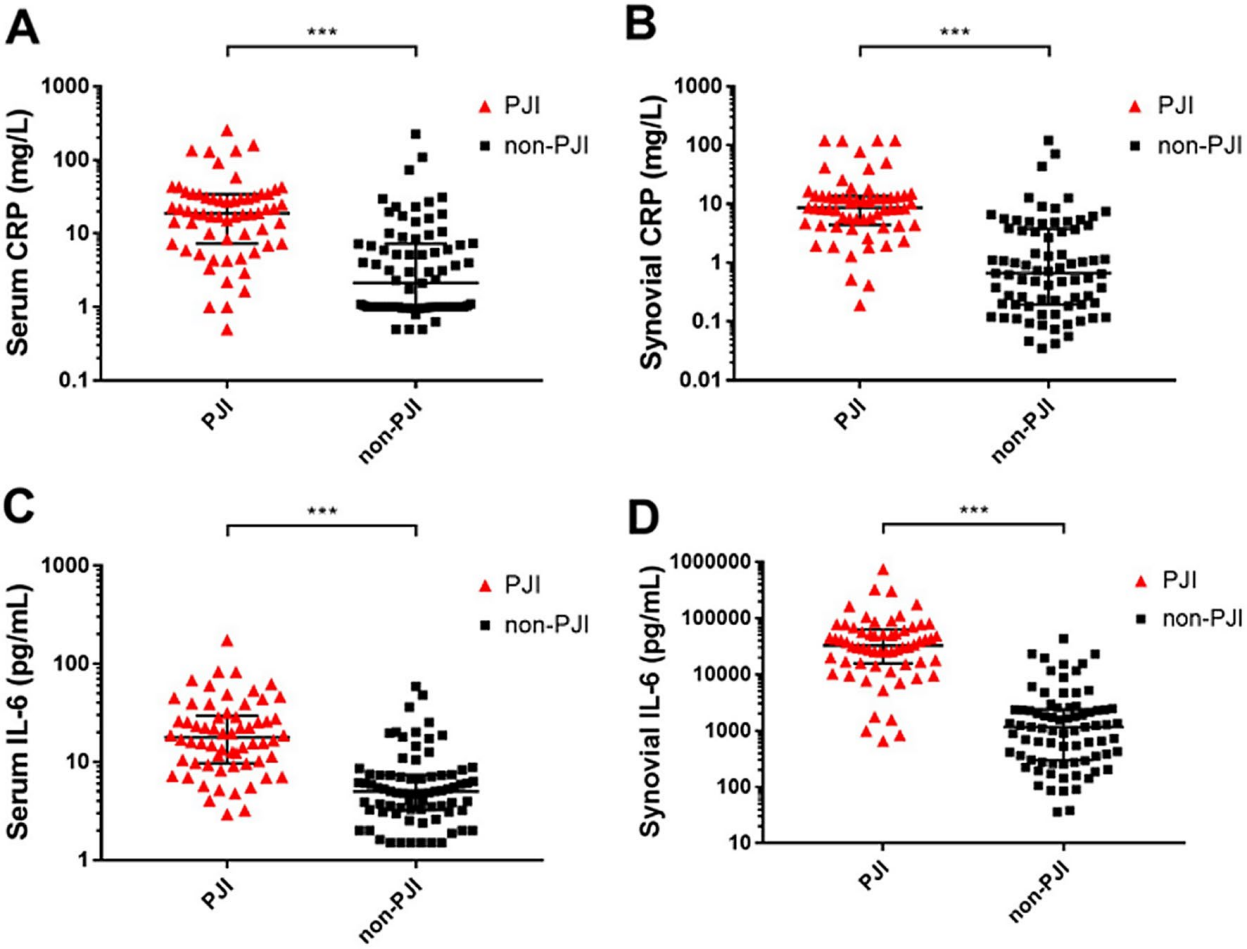
Scatter plots of the four indexes in the PJI and non-PJI groups. **A** CRP in serum; **B** CRP in synovial fluid; **C** IL-6 in serum; **D** IL-6 in synovial fluid; ***P-value<0.001; black horizontal line refers median; black bars refer to interquartile range

Scatter plots of the PJI and non-PJI groups are shown in Fig. 1. For the PJI group, the median (interquartile range; IQR) values of serum CRP (mg/L), synovial CRP (mg/L), serum IL-6 (pg/mL) and synovial IL-6 (pg/mL) were 18.79 (7.60–33.66), 8.62 (4.49–13.58), 17.80 (9.85–28.74) and 32,830.80 (16,163.81–60,259.27), respectively. For the non-PJI group, the median (IQR) values of serum CRP (mg/L), synovial CRP (mg/L), serum IL-6 (pg/mL) and synovial IL-6 (pg/mL) were 2.13 (1.00–7.20), 0.67 (0.19–3.72), 4.99 (3.26–7.33) and 1168.97 (303.09–2353.38), respectively. There were significant differences in each marker between the PJI and non-PJI groups.

**Fig. 2:**
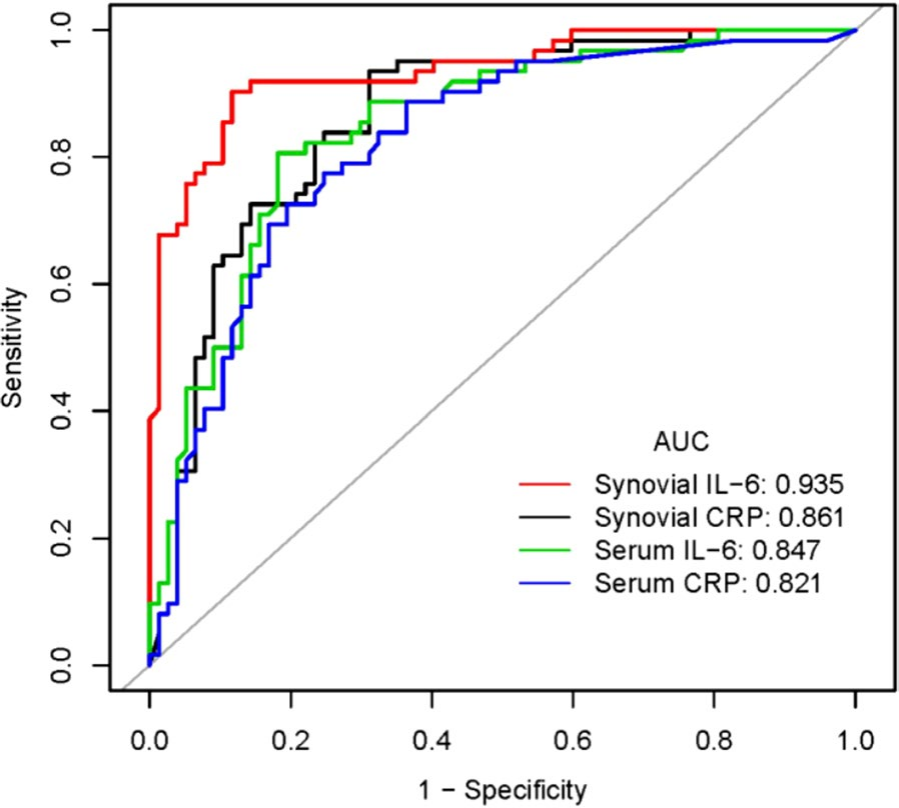
ROC curve of the four indexes for the diagnosis of PJI

The ROC curves showed that synovial IL-6 had the highest area under the curve (AUC), at 0.935, which was followed by the AUCs of synovial CRP, serum IL-6 and serum CRP: 0.861, 0.847 and 0.821, respectively (Fig. 2). The corresponding sensitivity, specificity, positive predictive value (PPV), and negative predictive value (NPV) of these markers are shown in Table 2.

**Table 2:** Results of the ROC curve analyses of the four indexes

Tests	AUC (95% CI)	Best threshold	Sensitivity	Specificity	PPV	NPV
Serum CRP (mg/L)	0.821 (0.750–0.892)	9.785	0.726	0.805	0.750	0.785
Serum IL-6 (pg/mL)	0.847 (0.782–0.912)	8.980	0.807	0.818	0.781	0.840
Synovial CRP (mg/L)	0.861 (0.799–0.924)	1.632	0.936	0.688	0.707	0.930
Synovial IL-6 (pg/mL)	0.935 (0.895–0.976)	6590.289	0.903	0.883	0.862	0.919

**Fig. 3:**
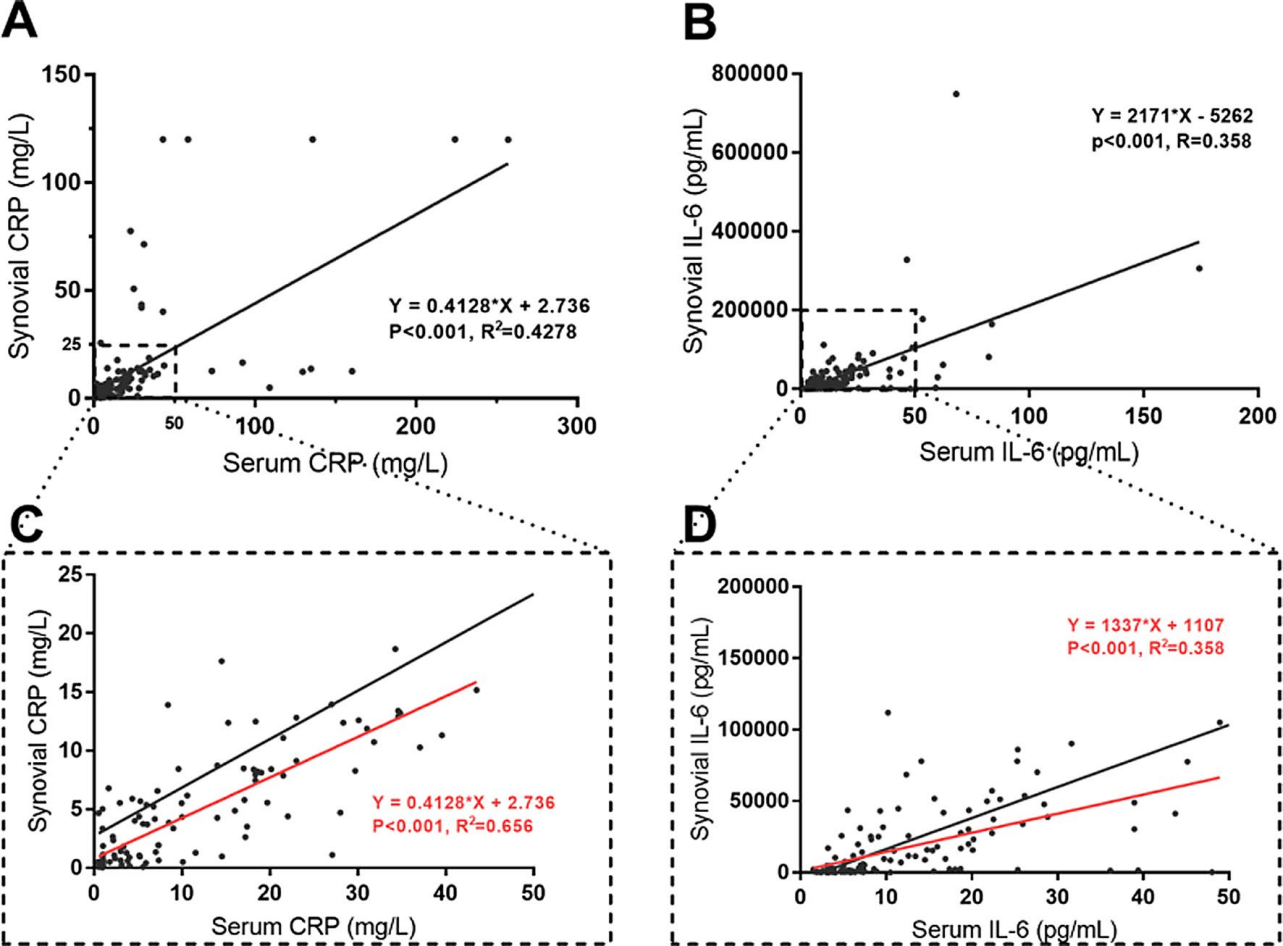
The distribution of CRP and IL-6 in serum and synovial fluid. **A** Distribution of CRP in serum and synovial fluid; **B** Distribution of IL-6 in serum and synovial fluid; **C** Zoomed image of the dotted rectangle area in **A**; **D** Zoomed image of the dotted rectangle area in **B**. Black line refers to the regression line by using the whole data for each marker; Red line refers to the regression line by using the data in rectangle area for each marker

The distributions of CRP and IL-6 in serum and synovial fluid are shown in Fig. 3. Both CRP and IL-6 showed discrete distributions, especially for the higher values of CRP and IL-6. The linear regression equation for CRP was *Y* = 0.4128*X + 2.736 (*P* < 0.0001, *R*^2^ = 0.4278), and the linear regression equation for IL-6 was *Y* = 2171*X − 5262 (*P* < 0.0001, *R*^2^ = 0.358). Both CRP and IL-6 showed poor goodness of fit. When excluded the outlier, only using the data in dotted rectangle area in Fig. 3, the linear regression equation for CRP was *Y* = 0.4128*X + 2.736 (*P* < 0.001, *R*^2^ = 0.656), and the linear regression equation for IL-6 was *Y* = 1337*X + 1107, (*P* < 0.001, *R*^2^ = 0.358). CRP showed a better goodness of fit, while the cost is the loss of 12.9% (18/139) data. Regarding IL-6, the goodness of fit remains at a poor level, and the cost is the loss of 6.5% (9/139) data.

**Fig. 4:**
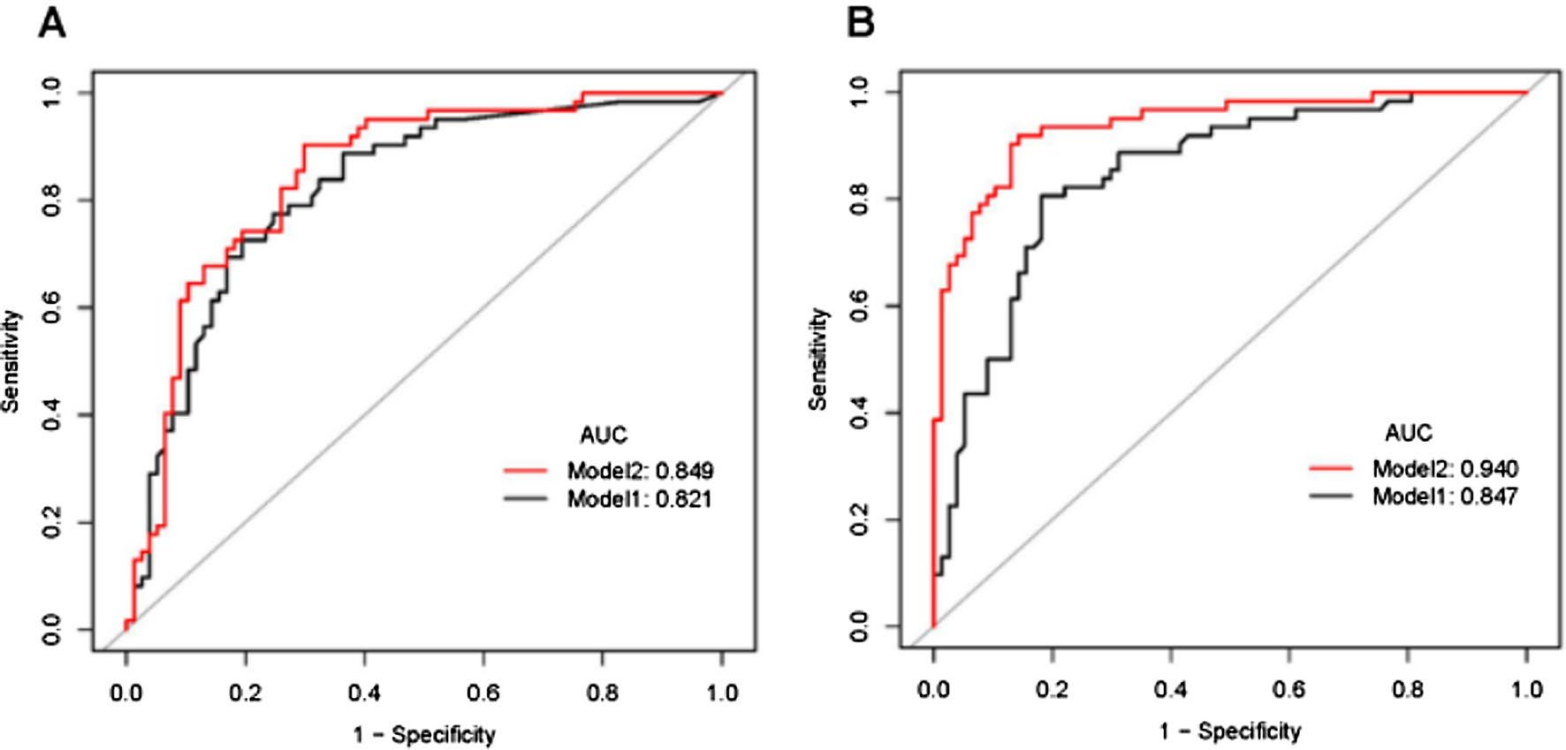
Comparisons of the prediction models for PJI diagnosis. **A** Comparisons of the prediction models between serum CRP (Model 1) and combination of serum and synovial CRP (Model 2) to predict PJI. **B** Comparisons of the prediction models between serum IL-6 (Model 1) and combination of serum and synovial IL-6 (Model 2) to predict PJI

We further investigated whether synovial CRP or IL-6 could add diagnostic value to the serum levels for the diagnosis of PJI. The prediction model for PJI using serum CRP/IL-6 alone was compared with that using the combination of serum and synovial CRP/IL-6. As shown in Fig. 4, when combining serum CRP and synovial CRP to diagnose PJI, the AUC was 0.849, which was only slightly higher than that associated with using serum CRP alone, which yielded an AUC of 0.821 (*P* = 0.021). In contrast, when combining serum IL-6 and synovial IL-6 to diagnose PJI, the AUC was increased to 0.940, which was significantly higher than that associated with using serum IL-6 alone (*P* < 0.001). The above findings illustrated that synovial CRP testing in addition to serum CRP results did not result in a better diagnostic accuracy, while synovial IL-6 was worthy of testing, even if a serum IL-6 result had already been obtained.

## Discussion

The main finding of the present study is the synovial IL-6 has the highest area under the curve (AUC), which was followed by the AUCs of synovial CRP, serum IL-6 and serum CRP, respectively. The relationship of C-reactive protein/interleukin-6 concentrations between serum and synovial fluid illustrated that synovial CRP testing in addition to serum CRP results did not result in a better diagnostic accuracy, while synovial IL-6 was worthy of testing, even if a serum IL-6 result had already been obtained.

CRP and interleukin-6 (IL-6) are two acute-phase reactive proteins. Both CRP and IL-6 are widely used in infection diagnosis. Based on our data, the best cutoff value of serum CRP was 9.785 mg/L, which was similar to the most widely used cutoff value (10 mg/L) [4, 12, 13]. The corresponding sensitivity and specificity were 0.726 and 0.805, respectively, which are within a reasonable range [14]. For synovial CRP, the best cutoff value was 1.632 mg/L. This result was similar to the results of Vanderstappen et al., who identified cutoff points for intra-articular CRP analysis of 1.8 mg/L and 2.8 mg/L [15]. Although our AUC of synovial CRP was not as high as 0.94, which was reported by a recent study [16], it was an acceptable value of 0.861 (0.799–0.924). Regarding IL-6, synovial IL-6 showed the highest AUC of 0.935 among the four indexes. Serum IL-6 showed an AUC of 0.847. This was consistent with the results of a recent meta-analysis, which showed that the pooled AUCs of serum and synovial IL-6 were 0.83 (95% CI 0.79–0.86) and 0.96 (95% CI 0.94–0.98), respectively [6]. The above findings reflected the reliability of our results.

Regarding the relationship of markers in serum and synovial fluid, Catterall et al. [11] postulated that diffusion of serum CRP into the joint could be responsible for an elevated synovial fluid CRP level when CRP is elevated systemically. Thus, there is a possibility that the level of these markers in serum could be used to predict the levels of these markers in synovial fluid. Based on our data, serum and synovial CRP had similar AUCs, which suggests that there is a possibility that the serum CRP level can be used to infer the level of CRP in the joint fluid. However, although there was a positive correlation between serum and synovial fluid CRP, the discrete distribution of CRP in Fig. 3 seemed to not support the postulation. The R-square value in the linear regression equation was 0.4278, which indicated a poor goodness of fit. Although CRP showed a better goodness of fit when excluded the outlier, only using the data in dotted rectangle area in Fig. 3, the cost is the loss of 12.9% (18/139) data. Thus, it does not seem feasible to infer the level of CRP in the synovial fluid from the serum level of CRP. A similar conclusion could be drawn from the results of IL-6.

Based on the finding that the concentrations of synovial markers are not closely dependent on those of serum markers, it is worth further identifying whether additional synovial fluid tests could contribute to serum tests to provide more accurate results. A prediction model using the combination of serum and synovial CRP was compared with another prediction model using serum CRP alone for PJI diagnosis. However, although there was a statistically significant difference between the two models (*P* = 0.021), the AUC of the combination model was 0.849, only slightly higher than that of the serum-alone model, which yielded an AUC of 0.821, and even lower than that of synovial CRP alone. Based on the above findings, we could conclude if serum CRP data are obtained, using either synovial CRP alone or in combination with serum would not offer a diagnostic advantage in the detection of PJIs. Tetreault et al. [17] put forward a similar viewpoint in their study. Regarding IL-6, using synovial IL-6 alone could result in better diagnostic accuracy than the serum tests. When combining serum IL-6 and synovial IL-6 to diagnose PJI, the AUC was increased to 0.940, which was slightly higher than that obtained when using synovial IL-6 alone and significantly higher than that obtained when using serum IL-6 alone. This indicates that serum IL-6 could not replace synovial IL-6 in the diagnosis of PJI.

Synovial fluid directly reflects the change in the local state of the joint, and in theory, synovial fluid tests could provide a more accurate diagnosis. However, because of the extra cost for each test and the limited volume of synovial fluid aspirated from some patients, choosing the proper synovial marker for further confirmation is crucial. Therefore, additional synovial CRP testing is not recommended for a patient who already has serum CRP results available for the diagnosis of PJI, while additional synovial IL-6 was worthy of testing even if there was already a serum IL-6 result.

There are some limitations to our study. First, the synovial test used frozen samples that were analyzed by ELISA, while serum tests used fresh blood samples that were analyzed by automatic machines in the Clinical Laboratory Center as part of our routine procedure. This may result in potential bias. However, the bias is holistic and does not affect their relationship trend. Second, due to the limitations of machines in the Clinical Laboratory Center, some of the levels of serum CRP and IL-6 were at the lower limit of detection. This may interfere with the analysis of the correlation between serum and synovial tests. However, this does not affect the analysis of its diagnostic efficacy by ROC curve. Finally, the sample size of this study was not large enough, and more studies with large sample sizes are needed in the future.

## Conclusion

In conclusion, synovial IL-6 has the highest diagnostic accuracy for PJI. The results of the other three tests, serum CRP, synovial CRP and serum IL-6, were similar. Inferring the level of CRP/IL-6 in the synovial fluid from the level of serum CRP/IL-6 was not feasible. For patients who already have results of serum CRP, additional synovial CRP testing would not result in better diagnostic accuracy. Synovial IL-6 was worthy of testing even if there was already a serum IL-6 result.

## Data Availability

We do not wish to share our data, because some of the patient’s data regarding individual privacy, and according to the policy of our hospital, the data could not be shared with others without permission.

## Abbreviations

CRP: C-reactive protein
IL-6: Interleukin-6
PJI: Periprosthetic joint infection
ESR: Erythrocyte sedimentation rate
WBC: White blood cell
MSIS: Musculoskeletal Infection Society
ELISA: Enzyme-linked immunosorbent assay
AUC: Area under curve
PPV: Positive predictive value
NPV: Negative predictive value
ROC: Receiver operating characteristic
WBC: White blood cell
BMI: Body mass index
